# Impact of COVID-19 containment measures on perceived health and health-protective behavior: a longitudinal study

**DOI:** 10.1038/s41598-023-50542-1

**Published:** 2024-01-03

**Authors:** Warner van Kersen, Myrna M. T. de Rooij, Lützen Portengen, Nekane Sandoval Diez, Inka Pieterson, Marjan Tewis, Jolanda M. A. Boer, Gerard Koppelman, Judith M. Vonk, Roel Vermeulen, Ulrike Gehring, Anke Huss, Lidwien A. M. Smit

**Affiliations:** 1 Institute for Risk Assessment Sciences (IRAS), Utrecht University, Utrecht, The Netherlands; 2 Centre for Nutrition, Prevention and Health Services, National Institute for Public Health and the Environment (RIVM), Utrecht, The Netherlands; 3 Department of Paediatric Pulmonology and Paediatric Allergology, University of Groningen, University Medical Center Groningen, Beatrix Children’s Hospital, Groningen, The Netherlands; 4 University of Groningen, University Medical Center Groningen, Groningen Research Institute of Asthma and COPD (GRIAC), Groningen, The Netherlands; 5 Department of Epidemiology, University of Groningen, University Medical Center Groningen, Groningen, The Netherlands; 6 Julius Centre for Health Sciences and Primary Care, University Medical Centre Utrecht, Utrecht, The Netherlands

**Keywords:** Environmental impact, Epidemiology

## Abstract

This longitudinal study aimed to assess the impact of COVID-19 containment measures on perceived health, health protective behavior and risk perception, and investigate whether chronic disease status and urbanicity of the residential area modify these effects. Participants (n = 5420) were followed for up to 14 months (September 2020-October 2021) by monthly questionnaires. Chronic disease status was obtained at baseline. Urbanicity of residential areas was assessed based on postal codes or neighborhoods. Exposure to containment measures was assessed using the Containment and Health Index (CHI). Bayesian multilevel-models were used to assess effect modification of chronic disease status and urbanicity by CHI. CHI was associated with higher odds for worse physical health in people with chronic disease (OR = 1.09, 95% credibility interval (CrI) = 1.01, 1.17), but not in those without (OR = 1.01, Crl = 0.95, 1.06). Similarly, the association of CHI with higher odds for worse mental health in urban dwellers (OR = 1.31, Crl = 1.23, 1.40) was less pronounced in rural residents (OR = 1.20, Crl = 1.13, 1.28). Associations with behavior and risk perception also differed between groups. Our study suggests that individuals with chronic disease and those living in urban areas are differentially affected by government measures put in place to manage the COVID-19 pandemic. This highlights the importance of considering vulnerable subgroups in decision making regarding containment measures.

## Introduction

Coronavirus disease 2019 (COVID-19), caused by severe acute respiratory syndrome coronavirus 2 (SARS-CoV-2), sparked an ongoing pandemic after it was first detected in Wuhan, China^1^. Apart from the direct health effects from the infection itself^2^, a range of indirect effects has emerged from the COVID-19 pandemic driven by fear of infection, stigma, anxiety and depression^3–7^. Likewise, the stringency of government measures to manage the outbreak has been shown to adversely affect health and wellbeing^8^. To date, most studies on indirect effects of the COVID-19 pandemic are cross-sectional in nature, while the stringency of containment measures has changed significantly over time^9^. Insights into the physical and mental health effects related to the stringency of containment measures issued by the government over time are, therefore, crucial in developing a better understanding of the indirect effects of COVID-19 associated with the containment measures in vulnerable groups.

People with identified risk factors for becoming seriously ill from COVID-19, such as diabetes, cardiovascular and respiratory disease^10^, were found to be more susceptible to these indirect effects in a study investigating the early phase of the pandemic^8^. To illustrate, a study in the United States among 1382 people with diabetes reported a substantive increase in both general and diabetes-related stress as well as social isolation, which significantly affected disease management^11^. Likewise, a survey among diabetes nurses in 27 European countries reported significant increases in physical and mental health issues in the population suffering from diabetes^12^. It has been shown that changes in the healthcare system of the Netherlands were associated with a decline in health status and an increase in psychological stress among patients with chronic cardiopulmonary disorders^13^. This suggests that indirect effects of the pandemic, mediated by containment measures, could be modified by pre-existing chronic disease. Alongside the vulnerability due to chronic conditions, an individual’s living environment could play a role in the indirect health effects of the pandemic. A study in the United States showed that cancer patients in urban areas, compared to rural areas, were more likely to practice COVID-19 protective behaviors^14^. This suggests that urbanicity of the residential area could be of importance in assessing the impact of the COVID-19 pandemic.

From September 2020 until November 2021, we performed a monthly online survey among in total 5420 participants of three Dutch cohort studies. The present study aimed to assess whether people with and without chronic disease (defined as: diabetes, cardiovascular disease, obesity, asthma or COPD) were differentially affected, in terms of perceived health and health-protective behavior, by the stringency of containment measures over time. In addition, we explored whether the impact of government stringency differed with urbanicity of the residential area.

## Materials and methods

### Study population and design

Participants for the “IMPACT” study were recruited from three existing Dutch prospective cohort studies, the Occupational and Environmental Health Cohort Study (AMIGO)^15^, the Livestock Farming and Neighboring Residents’ Health study (VGO)^16^, and the Prevention and Incidence of Asthma and Mite Allergy (PIAMA) study^17^. The design and sample selection for these studies have previously been described in detail. Briefly, AMGIO was designed to be representative of the general working population of the Netherlands. The cohort consists of 14,298 adults recruited between April 2011 and July 2012 from patient registries of 99 general practices (GP) spread across the country. Similarly, the 8772 adults participating in the VGO study were enrolled from the registries of 21 GPs in a livestock dense area in the south-east of the Netherlands in 2012. The PIAMA birth cohort (n = 3963) was established by enrolling pregnant women registered at one of 50 participating Dutch prenatal healthcare clinics between March 1996 and May 1997. A subset of 1912 participants of the PIAMA study could be contacted by email for the IMPACT study, resulting in a total of 24,982 eligible for the present study.

### Data collection

The Medical Research Ethics Committee (MERC) of the University Medical Centre Utrecht (UMCU) reviewed the study protocol (nr. 20/242) and ruled that official MERC approval was not required, because no invasive procedures were performed. All participants provided written informed consent before enrolment within the declaration of Helsinki framework. Participants were invited by post (VGO) or email (AMIGO and PIAMA). Each cohort had slightly different start dates and follow-up periods, from September 2020 to August 2021 for AMIGO, from December 2020 to August 2021 for PIAMA, and from December 2020 to October 2021 for VGO. Participation started with a baseline questionnaire assessing general characteristics and chronic disease status. Chronic disease was defined as having at least one of the following conditions: (1) asthma or Chronic Obstructive Pulmonary Disease (COPD), (2) cardiovascular disease, (3) diabetes mellitus, or (4) obesity (BMI > 30 kg/m^2^). Monthly follow-up questionnaires, sent at the beginning of each month, were used to collect information on perceived physical and mental health, COVID-19 related health-protective behavior and risk perception during the 4 weeks prior to completing the questionnaire date. All questionnaires were provided and completed through a (mobile) web-based application (COVapp). The questionnaires can be found in the supplementary material. To investigate differences between participants living in urban or rural areas, urbanicity of the residential area was obtained from the Dutch Central Bureau of Statistics (CBS) using the 4 digits of the postal code (AMIGO, VGO) or neighborhood (PIAMA) which were collected in 2014 (VGO), 2015 (AMIGO) and 2017–2018 (PIAMA)^18^. The five CBS categories of urbanicity were dichotomized, defining urban as > 1000 addresses per km^2^.

### Outcomes: perceived health, COVID-19 related health-protective behavior, and COVID-19 risk perception

We used 5-point physical and mental health scores ranging from excellent to poor from the monthly questionnaires as outcomes. As the category ‘poor’ was rarely chosen (Supplementary Figs. S1 and S2), we merged the two lowest categories (‘poor’ and ‘fair’), resulting in a 4-point ordinal scale for the analyses (excellent, very good, good, fair/poor). We investigated COVID-19 related protective behavior using the self-reported (1) average daily number of close contacts within the recommended social distancing of 1.5 m (excluding household members, categorized as 0–1, 2–5, 6–10, 11–20, > 20), (2) how often these contacts lasted longer than 10 min (< half, half or > half of close contacts) and (3) how often personal protective equipment (PPE) was used during these contacts (not at all, < half-, half-, > half of close contacts). We investigated COVID-19 related risk perception using perceived probability of (re)-acquiring COVID-19 (highly unlikely, unlikely, neutral, likely, highly likely) and perceived probability of becoming seriously ill from COVID-19 (highly unlikely, unlikely, neutral, likely, highly likely). Lastly, because healthcare availability could explain relationships between chronic disease status and perceived health, we explored two healthcare specific outcomes: healthcare avoidance in fear of acquiring COVID-19 in healthcare environments (does not describe me/my situation at all, does not describe me, neutral, describes me, describes me perfectly) and worrying about missed/postponed healthcare appointments (does not describe me/my situation at all, does not describe me, neutral, describes me, describes me perfectly).

### Exposure: stringency of COVID-19 containment measures

The stringency of the government measures to contain the COVID-19 outbreak was assessed using the Containment and Health Index (CHI) provided by the Oxford COVID-19 Government Response Tracker (OxCGRT)^19^. The CHI is an additive index ranging from 0 to 100, describing the severity of measures put in place by a government to manage the outbreak at any given date during the pandemic (https://ourworldindata.org/grapher/covid-containment-and-health-index). We used the CHI specific for the Netherlands as a standardized quantifier of the stringency of measures taken at the national level. Provided as a time series, day-to-day CHI values are based on 20 indicators divided in 3 categories: (1) Containment and closure (school closing, travel restrictions), (2) Economic response (income support, dept relief) and (3) Health systems (testing and vaccination policies). Monthly questionnaires assessed outcomes over a 4-week period prior to each questionnaire. Therefore, monthly averages of daily CHI values were used to quantify exposure to government measures in the month prior to each monthly questionnaire (e.g., March average CHI was assigned to April questionnaires).

### Statistical analysis

Data cleaning was performed by first removing participants missing baseline age, sex, BMI, urbanicity and chronic disease status. (n = 9; 0.2%), and then removing individual time points missing all outcome values (n = 14,275; 25%). To address missing data in the remaining dataset for the independent variables (at most 10.4% for a single variable), models were fitted on data imputed using the MICE package (version 3.14.15). Baseline age, sex, BMI, urbanicity and chronic disease status were imputed at the participant level (method = 2lonly.pmm). After 100 iterations of the imputation algorithm with default settings, 5 imputed datasets were generated. Outcome variables were included in the imputation procedure on record level but imputed outcome values were not used in subsequent analyses.

**Figure 1:**
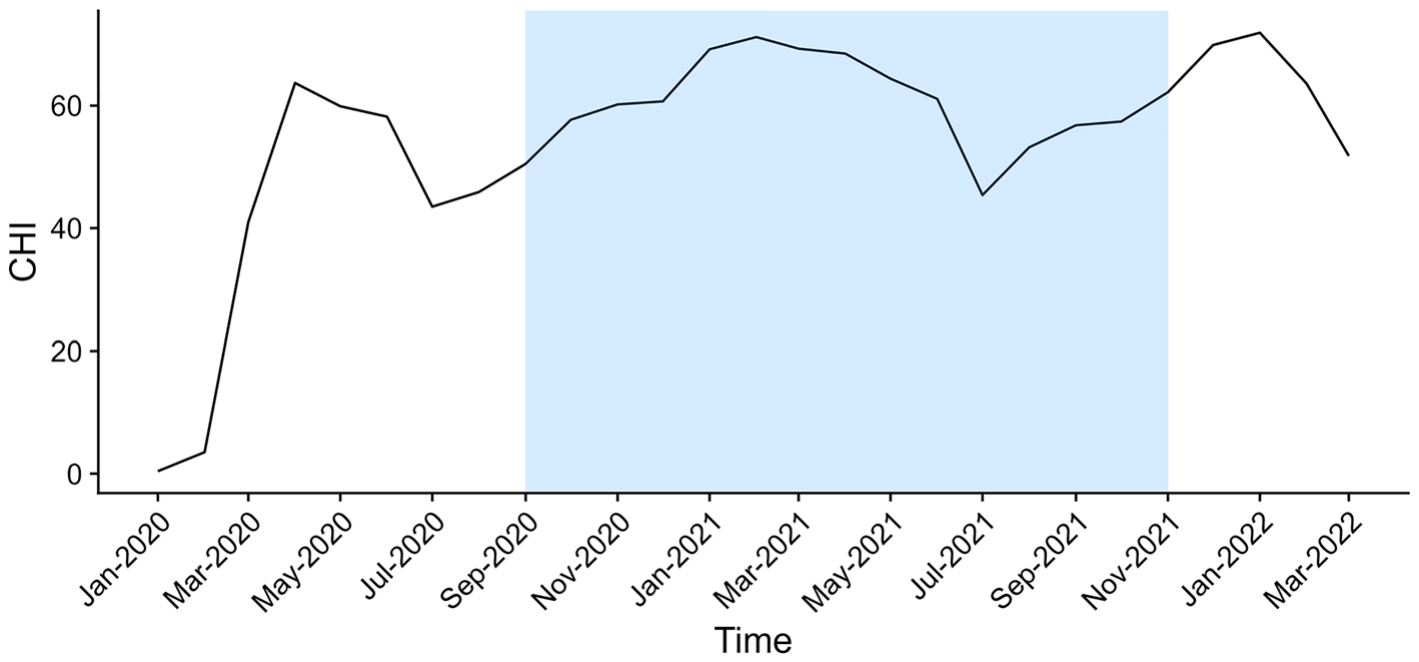
Monthly mean Containment and Health Index (CHI), depicting stringency of COVID-19 containment measures in the Netherlands over time. The study period is indicated by the shaded area.

Statistical analysis was performed using R (version 4.2.1) and RStudio^20^. We used a Bayesian multi-level model to accommodate the ordinal outcomes and time-series structure of the data as implemented in the BRMS package (version 2.18.0)^21^. Besides the default prior, number of iterations and warm-up (burn in), we used the “logit” link function and a first order autoregressive term to account for the correlation of observations within individuals over time. Chronic disease status, CHI (scaled to interquartile range, IQR) and urbanicity were included as explanatory variables. As the distribution pattern of the CHI over time showed a distinct seasonal pattern (Fig. 1), we included season as a potential confounder. This was done using sine and cosine functions of the observation date to estimate the amplitude and phase of the seasonal cycle. To increase precision of the estimates, models were additionally adjusted for age, sex, BMI and recruitment cohort (AMIGO, VGO, PIAMA).

Models with chronic disease—CHI and urbanicity—CHI interaction terms were used to assess whether the relationships between CHI and perceived health, COVID-19 related behavior and risk perception differ between people with and without a chronic disease and for urban versus rural populations. As notable differences in age and recruitment procedures exist between PIAMA and the other cohorts, a sensitivity analysis was performed excluding PIAMA participants. Likewise, a complete case analysis was performed in parallel to assess the impact of the imputation procedure. Results were expressed as odds ratios (OR) with 95% credibility intervals (CrI).

## Results

### Study population

A total of 5420 individuals participated, consisting of 3383 (62.4%) AMIGO, 1184 (21.8%) VGO and 853 (15.7%) PIAMA participants. The overall response rate was 22%, PIAMA had the highest response rate (44.6%) followed by AMIGO (23.7%), and VGO (13.5%). Nine participants (0.2%) were excluded as disease status and baseline characteristics for imputation were missing. Disease status was imputed for 512 individuals (9.5%). As a result, analyses were performed using data from 5411 individuals. An overview of the general characteristics of the study population can be found in Table 1. Participants with (compared to without) chronic disease were older (58.7y vs. 53.3y) had higher BMI (28.6 kg/m^2^ vs. 24.2 kg/m^2^), a higher proportion of females (55.8% vs. 52.8%) and a similar proportion of urban residents (53.2% vs. 52.8%). Within the chronic disease group, obesity was the most prevalent chronic condition (42.5%), followed by asthma or COPD (35.6%), cardiovascular disease (35.1%) and diabetes (14.2%). Overall, 17% of the participants reported a (suspected) SARS-CoV-2 (re-)infection before or during the study. As shown in Fig. 1, CHI ranged from 71.2 (most stringent, in February 2021) to 45.4 (least stringent, in July 2021).

**Table 1:** COVID-19 IMPACT study population characteristics per chronic disease status.

	Disease status known			Disease status imputed (N = 512)	Overall (N = 5411)
	No chronic disease (N = 3383)	Chronic disease (N = 1516)	*P* value		
Age [year]	53.2 [24.0, 78.0]	58.7 [24.0, 78.1]	< 0.001	55.9 [23.1, 78.0]	55.0 [23.1, 78.1]
Sex, Female	1889 (55.8%)	766 (50.5%)	< 0.001	279 (54.5%)	2934 (54.2%)
BMI [kg/m^2^]*	24.2 [15.6, 30.0]	28.6 [16.1, 56.7]	< 0.001	–	25.6 [15.6, 56.7]
Urbanicity of residential area			0.558		
< 1000 addresses/km^2^	1501 (44.4%)	693 (45.7%)		252 (49.2%)	2446 (45.2%)
> 1000 addresses/km^2^	1801 (53.2%)	800 (52.8%)		260 (50.8%)	2861 (52.9%)
Asthma or COPD	–	540 (35.6%)		–	540 (10.0%/11.0%)^†^
Diabetes	–	215 (14.2%)		–	215 (3.9%/4.4%)^†^
Cardiovascular disease	–	532 (35.1%)		–	532 (9.8%/10.9%)^†^
Obese (BMI > 30)	–	645 (42.5%)		–	645 (11.9%/13.2%)^†^
COVID-19 before or during study	592 (17.5%)	270 (17.8%)	0.823	56 (10.9%)	918 (17.0%)
Cohort			< 0.001		
AMIGO	2016 (59.6%)	1082 (71.4%)		285 (55.7%)	3383 (62.5%)
PIAMA	632 (18.7%)	140 (9.2%)		76 (14.8%)	848 (15.7%)
VGO	735 (21.7%)	294 (19.4%)		151 (29.5%)	1180 (21.8%)

Data are presented as mean [range] or n (%). *P*-values: Wilcoxon or chi^2^ test comparing known disease status groups. Disease status was imputed if baseline age, sex and urbanicity were available. *BMI = mass(kg)/(height (m))^2^. ^†^Percentages calculated for the overall total, and participants with known disease status (N = 5411/N = 4899).

Differences in baseline characteristics, and distribution of missing data, between cohorts can be found in supplementary Table S1. AMIGO and VGO were relatively similar in terms of mean age (61.0 vs. 59.6 years), percentage female sex (52.9% vs 50.5%) and BMI (26.1 vs. 25.7 kg/m^2^). PIAMA participants were younger (mean age 24.5 years), more often female (64.5%) and had a slightly lower average BMI (23.6 kg/m^2^). PIAMA was the most urbanized cohort (73.0%), followed by AMIGO (58.0%) and VGO (23.4%). As PIAMA participants were considerably younger, with exception of asthma, chronic disease was more prevalent in the other cohorts. The distributions of the outcomes over time can be found in supplementary Figs. S1–S9. A comparison between IMPACT study responders and non-responders using previously collected data, can be found in supplementary Table S2.

### Associations of CHI with perceived health

**Figure 2:**
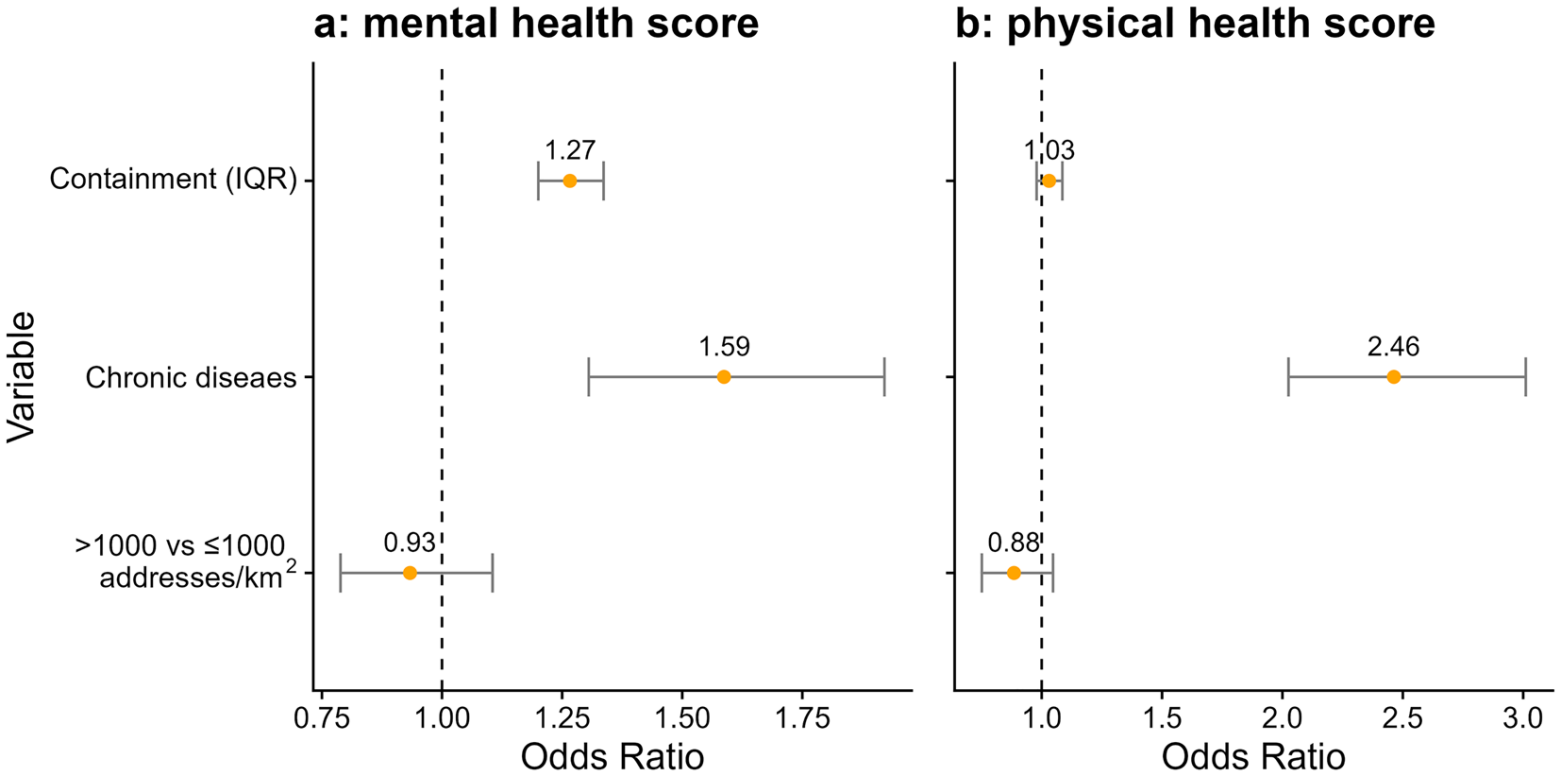
Bayesian multilevel main effect models for (**a**): perceived mental health score main effect model, (**b**): perceived physical health score main effect model. Models were adjusted for age, sex, BMI, recruitment cohort and season.

We investigated whether CHI was associated with perceived mental health scores, using main effects models (Fig. 2) and models with interaction terms (CHI x chronic disease and CHI x urbanicity; Table 2). In the main effects model, an IQR increase in CHI (IQR CHI = 11.5) was associated with increased odds of a worse mental health score (OR = 1.27, CrI = 1.20, 1.34). Likewise, participants with at least one chronic disease reported a worse mental health score (OR = 1.59, CrI = 1.31, 1.92). Urbanicity, however, was not found to be significantly associated with mental health (OR = 0.93, CrI = 0.79, 1.11). The interaction models showed no modification of the effect of CHI by chronic disease. However, the association of CHI with mental health was found to be more pronounced in urban (OR = 1.31 CrI = 1.23, 1.40) compared to rural areas (OR = 1.20, CrI = 1.13, 1.28).

**Table 2:** Interaction model results for Containment and Health Index with chronic disease and urbanization, including group specific effects.

	Chronic disease		Urbanization	
Outcomes (ordinal variables)	No	Yes	No	Yes
Mental health score	1.26 (1.19, 1.33)	1.28 (1.19, 1.39	**1.20 (1.13, 1.28)**	**1.31 (1.23, 1.40)**
Physical health score	**1.01 (0.95, 1.06)**	**1.09 (1.01, 1.17)**	1.03 (0.97, 1.10)	1.03 (0.97, 1.09)
N close contacts*	**0.55 (0.51, 0.59)**	**0.48 (0.44, 0.53)**	**0.57 (0.52, 0.61)**	**0.50 (0.47, 0.54)**
Close contacts > 10 min†	0.98 (0.92, 1.06)	1.01 (0.93, 1.11)	1.00 (0.92, 1.08)	0.99 (0.92, 1.07)
PPE usage during close contacts‡	1.71 (1.60, 1.84)	1.82 (1.66, 2.00)	**1.64 (1.51, 1.77)**	**1.83 (1.70, 1.98)**
Perceived risk of acquiring COVID-19	1.51 (1.41, 1.62)	1.48 (1.35, 1.62)	1.54 (1.45, 1.71)	1.44 (1.35, 1.55)
Perceived risk of severe COVID-19	**1.21 (1.13, 1.30)**	**1.50 (1.38, 1.64)**	1.30 (1.24, 1.40)	1.30 (1.21, 1.39)

Results are presented as odds ratios with 95% credible intervals. Abbreviations: CD = chronic disease. *Number of close contacts within 1.5 m as by Dutch covid legislation. †Fraction of close contacts with a duration longer than 10 min. ‡Fraction of close contacts in which personal protective equipment (PPE) was used. Bold: statistically significant interaction effects. Interaction effects were analyzed in separate models, adjusted for age, sex, BMI, chronic disease status, season and cohort.

Models with perceived physical health score as an outcome showed no statistically significant main effect of CHI, while chronic disease was associated with worse physical health (OR = 2.46, CrI = 2.03, 3.01). Models with interaction terms showed that the association with CHI was limited to participants with chronic disease (OR = 1.09, CrI = 1.01, 1.17) compared those without chronic disease (OR = 1.01, CrI = 0.95, 1.06). No association between urbanicity and physical health was observed (OR = 0.89, CrI = 0.75, 1.05) and no interaction between CHI and urbanicity was found.

### Associations of CHI with health-protective behavior

Main effect model results for COVID-related behavior outcomes can be found in supplementary Fig. S10. As expected, more stringent containment measures were associated with a lower number of close (within 1.5 m) personal contacts (OR = 0.53, CrI = 0.49, 0.56). Participants with chronic disease reported fewer contacts (on the ordinal scale) with persons within 1.5m compared to those without chronic disease (OR = 0.70, CrI = 0.56, 0.89). No main effect of urbanicity (OR = 0.92, CrI = 0.75, 1.13) on the number of close contacts was observed. Interaction models showed that the association of CHI tended to be stronger in participants with chronic disease (OR = 0.48, Cri = 0.44, 0.53) compared to those without (OR = 0.55, Cri = 0.51, 0.59). Likewise, the association of CHI was found to be stronger in urban (OR = 0.50, CrI = 0.47, 0.54) compared to rural areas (OR = 0.57, CrI = 0.52, 0.61).

No association between CHI and close contact duration was observed (OR = 0.99, CrI = 0.93, 1.06). Similarly, chronic disease and urbanicity were not associated with contact duration. In addition, no interaction was found between CHI and chronic disease or urbanicity in relation to close contact duration. On the other hand, odds for PPE usage during close contacts were shown to increase with increasing CHI (OR = 1.75, CrI = 1.63, 1.87). Chronic disease status and urbanicity were not associated with PPE usage during close contacts. However, a borderline significant interaction term suggested that the association of CHI with PPE usage was more pronounced in participants with chronic disease (Table 2). Likewise, the effect of CHI on PPE usage was found to be stronger in urban areas.

### Associations of CHI with COVID-19 risk perception

Perceived probability of acquiring COVID-19 was found to be positively associated with CHI and chronic disease, but no association with urbanicity was identified (Supplementary Fig. S11A,B). A borderline significant interaction term suggested that the association with CHI was more pronounced in rural (OR = 1.54, CrI = 1.45, 1.71) compared to urban areas (OR = 1.44 CrI = 1.35, 1.55). No interaction between chronic disease and CHI was found. Models with outcome ‘perceived probability of becoming seriously ill from COVID-19’ showed positive associations with a higher CHI (OR = 1.30, CrI = 1.22, 1.38). Likewise, chronic disease status (OR = 5.81, CrI = 4.86, 6.98) was strongly associated with perceived risk of severe COVID-19. A significant interaction term (OR = 1.24, CrI = 1.13, 1.36) showed that the association with CHI was stronger in people with chronic disease. No association between urbanicity of the residential area and perceived probability of severe illness was found.

### Associations of CHI with healthcare avoidance

Models investigating healthcare avoidance (due to fear of acquiring COVID-19 in healthcare environments, Supplementary Fig. S11C,D) suggested that a higher CHI was associated with lower odds for healthcare avoidance (OR = 0.94, CrI = 0.88, 1.01) but this association was not statistically significant. Chronic disease was associated with higher odds for healthcare avoidance (OR = 1.26 CrI = 1.12, 1.53). The model with outcome ‘worrying about missed or postponed healthcare appointments’ showed that chronic disease (OR = 1.60, CrI = 1.40, 1.82) but not CHI was associated with higher odds for worrying.

### Sensitivity analyses

As the PIAMA cohort differs substantially from both AMIGO and VGO in terms of age and recruitment procedure, we performed a sensitivity analysis to investigate whether this influenced our results. To this end, the interaction models (between CHI and chronic disease as well as CHI and urbanicity) were re-analyzed using data from AMIGO and VGO participants only, thus excluding PIAMA participants. Results of these models (supplementary Table S3) show that cohort differences did not significantly affect our results. A comparison of the results with a complete case analysis can be found in supplementary Tables S4 and S5. Besides the narrower credibility intervals, indicating that the multiple imputation analysis was more efficient, no apparent differences were found.

## Discussion

In this study we investigated whether the intensity of national COVID-19 containment measures, expressed by CHI in the Netherlands, differentially affected people with or without a chronic disease and residents of urban versus rural areas. We found that associations of CHI with perceived health, health protective behavior, and risk perception were more pronounced in participants with a chronic disease and residents of urban areas. Mental health decreased with increasing CHI. While this association was not dependent on chronic disease status, it was shown to be more pronounced in residents of urban areas. This decrease in mental health, could be (partly) explained by our finding that CHI was associated with increased risk perception for COVID-19 infection and severity. The relationship between CHI and perceived probability of a severe COVID-19 infection was found to be stronger among those suffering from a chronic disease. Additionally, participants with a chronic disease reported worsening physical health with increasing CHI. This was not seen in participants without a chronic disease, indicating that chronic disease confers a predisposition to worsening physical health during peaks in stringency of containment measures.

These findings are in line with cross-sectional reports of worse health in chronically diseased individuals during the pandemic^12,22,23^. Our longitudinal analyses showed that this association is related to fluctuations in the stringency of containment measures over time. It has been suggested that the decline in health is related to unavailable or inaccessible healthcare^13,22,24^. In this study, we show that healthcare avoidance (due to fear of acquiring COVID-19 at a healthcare facility) decreased with increasing CHI. This suggests that containment measures provide a sense of security in relation to the use of healthcare services aiding the continuation of regular healthcare. However, having a chronic disease was associated with healthcare avoidance. Thus, ensuring and propagating patient safety in healthcare environments is crucial during pandemics.

Individuals with a chronic disease reported fewer close contacts (within 1.5 m) during which they used PPE more often. This increase in health protective behavior may be explained by increased COVID-19 risk perception which is supported by our finding that chronic disease was strongly associated with perceiving an increased probability of infection with SARS-CoV-2 and severe COVID-19. Other factors related to chronic disease (e.g. decreased mobility) could also play a role. Similarly, living in an urban area (compared to rural) was associated with fewer contacts during which PPEs were used more often. Urbanicity, however, was not associated with perceiving an increased probability of infection or severe disease. This suggests that the inclination of urban residents towards health protective behavior is driven by other factors like social pressure, which is in line with the Dutch public debate during the pandemic, stressing a (presumed) elevated infection risk in cities^25^. These findings could also be a reflection of the more profound change in day-to-day life in cities (empty streets, closed shops) during lockdown. We showed that the associations of CHI (which incorporates group size restrictions and PPE policies) on the number of contacts and PPE usage were more pronounced in individuals with chronic disease and urban residents respectively, indicating an increased inclination to adhere to containment measures in both groups.

Evidence on the role of urbanicity in mental health is inconclusive. There are reports of beneficial effects of living in a rural area^26^, while other studies find no associations between mental health and living in urban areas^27^. A recent study assessing the role of housing environment on mental health during the pandemic reported no associations with urbanicity and mental health indicators^28^. However, they did report that lacking access to an outdoor space (e.g. garden or balcony) was associated with worse mental health outcomes during lockdown. Likewise, an Italian study reported an association between living in apartments smaller than 60m^2^ and increased risk of depressive symptoms in students^29^. This may potentially explain that we found a stronger association with CHI in urban areas, where typically homes are smaller and without private outdoor spaces. Also of importance are differences in available services and amenities between urban and rural areas, resulting in more pronounced changes in day-to-day life in urban areas during lockdown. We showed that VGO participants, mainly living in rural municipalities, reported better mental health compared to AMIGO participants, who are more evenly distributed along the urban–rural gradient. As our models are corrected for urbanicity, this indicates that other regional factors could play a role. The fact that VGO participants reported a higher perceived probability of acquiring COVID-19 is potentially explained by the fact that most VGO participants live in the province of Noord-Brabant, which was the epicenter of the initial start of the epidemic in the Netherlands^30^. Our finding that older individuals reported better mental health than younger participants, is in line with reports of pandemic-related mental health issues in young people^31^, which can be explained by differences in coping strategies and support structures between children, adolescents and adults.

Limitations of this study include the response rate of 22%, conferring potential influence of non-response bias. However, comparing study sample characteristics with the source population revealed relatively minor differences in age, sex and smoking habits. Another limitation are the substantial differences between cohorts. These differences, however, mainly involve differences in recruitment and younger age of PIAMA participants (a birth cohort). On the other hand, AMIGO and VGO were recruited in an identical manner amongst adult registered at general practices resulting in similar personal characteristics. A sensitivity analysis without PIAMA showed that these differences did not significantly influence our results. Another limitation is the correlation between CHI and time. By taking the multi-level structure of our data into account, we were able to analyze individual outcome trajectories. However, as a result of adjustment for correlation over time between observations, our multi-level model underestimates true associations with CHI. There is a risk of residual confounding due to unmeasured time-varying factors which could be amplified by the subjective nature of questions on perceptions and behaviors. The absence of a baseline health score measurement before the onset of the pandemic prevents comparison to a situation without any containment measures.

Main strengths of this study include the longitudinal design combined with the use of CHI as a standardized assessment of exposure to containment measures, enabling estimation of the effect of CHI while controlling for individual confounders that are stable over time. Another strength is our use of multiple imputation to address missing data, resulting in more precise effect estimates than more basic approaches like complete case analysis or mean imputation. The unique challenges faced (e.g., questionnaire app development, data protection clearance) in setting up this study efficiently during the initial days of the pandemic emphasize the need for ‘ready to go’ research frameworks that are easy to deploy in future public health crises.

## Conclusion

Our study suggests that the stringency of government measures, put in place to manage the outbreak in the Netherlands, differentially affected people with chronic disease and residents of urban areas, emphasizing the importance of considering vulnerable subgroups in decision making about containment measures in public health crises.

### Supplementary Information

Supplementary Information 1.

Supplementary Information 2.

## Data Availability

The data underlying this article will be shared with researchers who provide a methodologically sound proposal on request to the corresponding author.
