# Supplementary material for: Impact of COVID-19 containment measures on perceived health and health-protective behavior: a longitudinal study
Source: Sci Rep. 2024 Jan 3;14:419. doi: 10.1038/s41598-023-50542-1 (PMC10764319; doi:10.1038/s41598-023-50542-1)
Supplement: Supplementary file 1 — Supplementary Information 1. [file 41598_2023_50542_MOESM1_ESM.docx]

**Impact of the COVID-19 pandemic on perceived health and health-protective behavior in individuals with and without a chronic disease: a longitudinal study.**

**Supplementary material**

Warner van Kersen, Myrna M. T. De Rooij, Lützen Portengen, Nekane Sandoval Diez, Inka Pieterson, Marjan Tewis, Jolanda M.A. Boer, Gerard Koppelman, Judith M. Vonk, Roel Vermeulen, Ulrike Gehring, Anke Huss*, Lidwien A.M. Smit*

* these two authors contributed equally

Version: 20231112

**Questionnaire Q1. Baseline Questionnaire**

I. What is your date of birth?

II. What is your sex?

0. Male

1. Female

III. What is your current weight? (kg)

IIV. What is your height? (cm)

V. For each of the conditions below, please indicate whether you have (had) them.

a. Heart attack (0=no,1=yes)

b. Narrowed arteries legs (0=no,1=yes)

c. Stroke or TIA (0=no,1=yes)

d. Other heart condition (0=no,1=yes)

e. Lung disease asthma, COPD, chronic bronchitis (0=no,1=yes)

f. Diabetes Mellitus (0=no,1=yes)

**Questionnaire Q2. Monthly Questionnaires**

I. How do you rate your physical health? (in relation to the past 4 weeks)

1. Poor

2. Fair

3. Good

4. Very good

5. Excellent

II. How do you rate your mental health? (in relation to the past 4 weeks)

1. Poor

2. Fair

3. Good

4. Very good

5. Excellent

III. How many different people came within 1.5m of you? On average per day, excluding household members.

1. 0-1
2. 2-5
3. 6-10
4. 11-20
5. >20

IV. In how many cases did these contacts last longer than 10 minutes?

1. In less than half of the cases
2. In approximately half the cases
3. In more than half of the cases

V. Did you use any personal protective equipment during these contacts? e.g. facemask, gloves or a screen?

1. No
2. In less than half the cases
3. In approximately half the cases
4. in more than half of the cases

VI. How likely do you believe it to be that you will get Coronavirus/COVID-19 (again)?

1. Very unlikely
2. Somewhat unlikely
3. Neutral
4. Somewhat likely
5. Very likely

VII. If you were to get Coronavirus/COVID-19, how likely do you believe it to be that you will become seriously ill

1. Very unlikely
2. Somewhat unlikely
3. Neutral
4. Somewhat likely
5. Very likely

IIX. Please indicate how well the following statement describes your behaviour: I avoid healthcare because I am afraid of getting Coronavirus/COVID-19 that way.

1. Doesn’t describe me at all
2. Does not describe me
3. Neutral
4. Describes me
5. Describes me exactly

IIX. Please indicate how well the following statement describes your behaviour: I worry about the impact of my missed/postponed healthcare appointments.

1. Doesn’t describe me at all
2. Does not describe me
3. Neutral
4. Describes me
5. Describes me exactly

Note: The Likert scales used in questions I and II were inverted, to range from 5 (excellent) to 1 (poor), for analytical purposes.
