# Supplementary material for: Impact of COVID-19 containment measures on perceived health and health-protective behavior: a longitudinal study
Source: Sci Rep. 2024 Jan 3;14:419. doi: 10.1038/s41598-023-50542-1 (PMC10764319; doi:10.1038/s41598-023-50542-1)
Supplement: Supplementary file 2 — Supplementary Information 2. [file 41598_2023_50542_MOESM2_ESM.docx]

**Impact of COVID-19 containment measures on perceived health and health-protective behavior: a longitudinal study**

**Supplementary tables and figures**

Warner van Kersen, Myrna M. T. De Rooij, Lützen Portengen, Nekane Sandoval Diez, Inka Pieterson, Marjan Tewis, Jolanda M.A. Boer, Gerard Koppelman, Judith M. Vonk, Roel Vermeulen, Ulrike Gehring, Anke Huss*, Lidwien A.M. Smit*

* these two authors contributed equally

Supplementary table S1: COVID-19 IMPACT study population characteristics per cohort, including missing data.

|  | **AMIGO (N=3,383)** | **PIAMA (N=848)** | **VGO (N=1,180)** | **P-value** |
| --- | --- | --- | --- | --- |
| **Age (y)** | 61.0 [39.0, 76.8] | 24.6 [23.1, 25.3] | 59.6 [26.2, 78.1] | <0.001 |
| Missing | 0 (0%) | 2 (0.2%) | 0 (0%) |  |
| **Sex, female** | 1,788 (52.9%) | 548 (64.6%) | 598 (50.7%) | <0.001 |
| Missing | 0 (0%) | 2 (0.2%) | 0 (0%) |  |
| **BMI (kg/m2)*** | 26.1 [16.1, 56.7] | 23.6 [15.6, 48.5] | 25.7 [16.8, 42.9] | <0.001 |
| Missing | 320 (9.5%) | 84 (9.8%) | 171 (14.4%) |  |
| **Urbanicity** |  |  |  | <0.001 |
| <1000 addresses/km^2^ | 1,422 (42.0%) | 165 (19.5%) | 859 (72.6%) |  |
| >1000 addresses/km^2^ | 1,961 (58.0%) | 623 (73.5%) | 277 (23.4%) |  |
| Missing | 0 (0%) | 60 (7.0%) | 48 (4.1%) |  |
| **Chronic disease** | 1,082 (32.0%) | 140 (16.5%) | 294 (24.9%) | <0.001 |
| Asthma or COPD | 347 (10.3%) | 102 (12.0%) | 91 (7.7%) | 0.0189 |
| Diabetes | 174 (5.1%) | 3 (0.4%) | 38 (3.2%) | <0.001 |
| Cardiovascular disease | 408 (12.1%) | 4 (0.5%) | 120 (10.2%) | <0.001 |
| Obese (BMI>30) | 493 (14.6%) | 43 (5.1%) | 109 (9.2%) | <0.001 |
| Missing | 285 (8.4%) | 76 (9.0%) | 151 (12.8%) |  |
| **COVID-19 before or during study** | 491 (14.5%) | 211 (24.9%) | 216 (18.3%) |  |
| Missing | 0 (0.00%) | 0 (0.00%) | 0 (0.00%) |  |
| Data are presented as mean [range] or n (%). P-value: Kruskal-Wallis or chi^2^ test. *BMI=mass(kg)/(height (m))^2^. | | | | |

Supplementary Table S2: COVID-19 IMPACT characteristics of responders and non-responders

|  | **IMPACT Sample**  **(N=5,420)** | **Non-responders**  **(N=19,216)** | **Source Population**  **(N=24,636)** | **P value*** |  |
| --- | --- | --- | --- | --- | --- |
| **Cohort** |  |  |  |  |  |
| PIAMA | 853 (15.7%) | 1,059 (5.5%) | 1,912 (7.8%) | <0.001 |  |
| AMIGO | 3,383 (62.4%) | 10,915 (56.8%) | 14,298 (58.0%) |  |  |
| VGO | 1,184 (21.8%) | 7,242 (37.7%) | 8,426 (34.2%) |  |  |
| **Age** |  |  |  |  |  |
| Mean (SD) | 55.1 (15.8) | 56.7 (13.3) | 56.4 (13.9) | <0.001 |  |
| Median (Q1,Q3) | 59.0 (48.5,67.3) | 58.0 (49.0,67.0) | 58.2 (48.9, 67.0) |  |  |
| Min-Max | 24.0 – 79.2 | 24.3 – 79.4 | 24.0 – 79.4 |  |  |
| Missing | 527 | 0 | 527 |  |  |
| **Sex** |  |  |  |  |  |
| Male | 2,240 (45.8%) | 8,687 (45.2%) | 10,927 (45.3%) | 0.432 |  |
| Female | 2,647 (54.2%) | 10,528 (54.8%) | 13,175 (54.7%) |  |  |
| Missing | 533 | 1 | 534 |  |  |
| **Smoking Status** |  |  |  |  |  |
| Never | 2,055 (45.2%) | 5,693 (42.8%) | 7,748 (43.4%) | <0.001 |  |
| Current smoker | 504 (11.1%) | 2,091 (15.7%) | 2,595 (14.5%) |  |  |
| Former smoker | 1,992 (43.8%) | 5,509 (41.4%) | 7,501 (42.0%) |  |  |
| Missing | 869 | 5,923 | 6,792 |  |  |
| *Linear model ANOVA or Pearson’s chi-squared test | | | | | |

Supplementary table S3: Interaction effects of Containment and Health Index with chronic disease and urbanization, all three IMPACT cohorts combined and sensitivity analysis excluding PIAMA participants.

|  | Interaction CHI x CD | | Interaction CHI x Urban | |
| --- | --- | --- | --- | --- |
| Outcomes (ordinal variables) | IMPACT study (Pooled) | Excluding PIAMA | IMPACT study (Pooled) | Excluding PIAMA |
| Mental health score | 1.02 (0.94,1.10) | 1.05 (0.98,1.14) | **1.09 (1.02,1.27)** | 1.05 (0.98,1.13) |
| Physical health score | **1.08 (1.00,1.17)** | 1.07 (0.98-1.15) | 1.00 (0.93,1.07) | 1.01 (0.94,1.09) |
| N close contacts* | **0.89 (0.81,0.97)** | **0.87 (0.79,0.96)** | **0.88 (0.81,0.96)** | **0.88 (0.81,0.96)** |
| Close contacts >10min† | 1.03 (0.94,1.13) | 1.04 (0.94,1.15) | 1.00 (0.92,1.09) | 0.98 (0.89,1.08) |
| PPE usage during close contacts‡ | 1.06 (0.98,1.17) | 1.02 (0.94,1.12 | **1.12 (1.03,1.22)** | **1.20 (1.10,1.31)** |
| Probability of acquiring COVID-19 | 0.98 (0.89,1.08) | 0.98 (0.88,1.08) | 0.92 (0.84,1.00) | 0.93 (0.85,1.02) |
| Probability of severe COVID-19 | **1.24 (1.13,1.36)** | **1.22 (1.11,1.35)** | 1.00 (0.92,1.08) | 1.08 (0.99,1.17) |
| Results are presented as odds ratios with credible intervals. Abbreviations: CD = chronic disease. * Number of close contacts within 1.5m as by Dutch covid legislation. † Fraction of close contacts with a duration longer than 10 minutes. ‡ Fraction of close contacts in which personal protective equipment (PPE) was used. Bold: statistically significant interaction effects. Models were adjusted for age, sex, BMI, chronic disease status, season and cohort. | | | | |

Supplementary table S4: Interaction effects of Containment and Health Index with chronic disease and urbanization, complete case analysis and multiple imputation results.

|  | Interaction CHI x CD | | Interaction CHI x Urban | | |  |
| --- | --- | --- | --- | --- | --- | --- |
| Outcomes (ordinal variables) | Complete case | Multiple imputation | Complete case | Multiple imputation |  |  |
| Mental health score | 1.03 (0.95,1.12 | 1.02 (0.94,1.10) | **1.09 (1.01,1.18)** | **1.09 (1.02,1.27)** |  |  |
| Physical health score | **1.07 (1.00,1.17** | **1.08 (1.00,1.17)** | 1.01 (0.94,1.08) | 1.00 (0.93,1.07) |  |  |
| N close contacts* | **0.89 (0.81,0.98)** | **0.89 (0.81,0.97)** | 0.96 (0.88,1.04) | **0.88 (0.81,0.96)** |  |  |
| Close contacts >10min† | 1.03 (0.94,1.14) | 1.03 (0.94,1.13) | 1.01 (0.94,1.09) | 1.00 (0.92,1.09) |  |  |
| PPE usage during close contacts‡ | **1.12 (1.00,1.23** | 1.06 (0.98,1.17) | 0.93 (0.86,1.00) | **1.12 (1.03,1.22)** |  |  |
| Probability of acquiring COVID-19 | 0.98 (0.88,1.09) | 0.98 (0.89,1.08) | 0.90 (0.81,1.00) | 0.92 (0.84,1.00) |  |  |
| Probability of severe COVID-19 | **1.33 (1.20,1.48)** | **1.24 (1.13,1.36)** | 0.99 (0.90,1.10) | 1.00 (0.92,1.08) |  |  |
| Results are presented as odds ratios with credible intervals. Abbreviations: CD = chronic disease. * Number of close contacts within 1.5m as by Dutch covid legislation. † Fraction of close contacts with a duration longer than 10 minutes. ‡ Fraction of close contacts in which personal protective equipment (PPE) was used. Bold: statistically significant interaction effects. Models were adjusted for age, sex, BMI, chronic disease status, season and cohort. | | | | | |  |
|  |  |  |  |  |  |  |

Supplementary table S5: Main effect models results for all IMPACT study outcomes, complete case analysis and multiple imputation results.

|  | Complete case | Multiple imputation |  |
| --- | --- | --- | --- |
| **Outcomes (ordinal)** and predictors | OR + (95%CrI) | OR + (95%CrI) |  |
| **Mental health score** |  |  |  |
| Chronic disease | 1.68 (1.34,2.10) | 1.59 (1.31,1.92) |  |
| Containment | 1.29 (1.22,1.36) | 1.27 (1.20,1.34) |  |
| >1000 addresses/km2 | 0.93 (0.77,1.12) | 0.93 (0.79,1.11) |  |
| **Physical health score** |  |  |  |
| Chronic disease | 2.51 (2.01,3.11) | 2.46 (2.03,3.01) |  |
| Containment | 1.02 (0.97,1.08) | 1.03 (0.98,1.09) |  |
| >1000 addresses/km2 | 0.89 (0.74,1.06) | 0.88 (0.75,1.05) |  |
| **N close contacts*** |  |  |  |
| Chronic disease | 0.66 (0.51,0.85) | 0.70 (0.56,0.89) |  |
| Containment | 0.51 (0.48,0.55) | 0.53 (0.49,0.56) |  |
| >1000 addresses/km2 | 0.89 (0.71,1.12) | 0.92 (0.75,1.13) |  |
| **Close contacts >10 min**† |  |  |  |
| Chronic disease | 0.91 (0.76,1.10) | 0.96 (0.81,1.15) |  |
| Containment | 1.00 (0.93,1.08) | 0.99 (0.93,1.06) |  |
| >1000 addresses/km2 | 0.93 (0.79,1.09) | 0.96 (0.83,1.12) |  |
| **PPE usage during close contacts‡** |  |  |  |
| Chronic disease | 1.05 (0.86,1.28) | 1.03 (0.87,1.22) |  |
| Containment | 2.00 (1.85,2.16) | 1.75 (1.63,1.87) |  |
| >1000 addresses/km2 | 0.94 (0.80,1.12) | 0.94 (0.82,1.08) |  |
| **Probability of aquiring COVID-19** |  |  |  |
| Chronic disease | 1.30 (1.06,1.60) | 1.35 (1.15,1.58) |  |
| Containment | 1.55 (1.44,1.67) | 1.50 (1.41,1.60) |  |
| >1000 addresses/km2 | 0.94 (0.78,1.12) | 0.97 (0.85,1.12) |  |
| **Probability of severe COVID-19** |  |  |  |
| Chronic disease | 8.87 (6.89,11.41) | 5.81 (4.86,6.98) |  |
| Containment | 1.36 (1.27,1.46) | 1.30 (1.22,1.38) |  |
| >1000 addresses/km2 | 1.04 (0.84,1.29) | 1.04 (0.90,1.21) |  |
| **Healthcare-avoidance** |  |  |  |
| Chronic disease | 1.55 (1.23,1.96) | 1.26 (1.12,1.42) |  |
| Containment | 0.89 (0.73,1.09) | 0.94 (0.88,1.01) |  |
| >1000 addresses/km2 | 0.78 (0.67,0.91) | 0.88 (0.80,0.97) |  |
| **Worries about missed healthcare** |  |  |  |
| Chronic disease | 1.80 (1.46,2.22) | 1.60 (1.40,1.82) |  |
| Containment | 0.84 (0.72,0.98) | 0.94 (0.88,1.01) |  |
| >1000 addresses/km2 | 0.97 (0.81,1.15) | 0.96 (0.87,1.06) |  |
| Results are presented as odds ratios with credible intervals. * Number of close contacts within 1.5m as by Dutch covid legislation. † Fraction of close contacts with a duration longer than 10 minutes. ‡ Fraction of close contacts in which personal protective equipment (PPE) was used. Variables were mutually adjusted for. Models were adjusted for age, sex, BMI, chronic disease status, season and cohort. | | | |


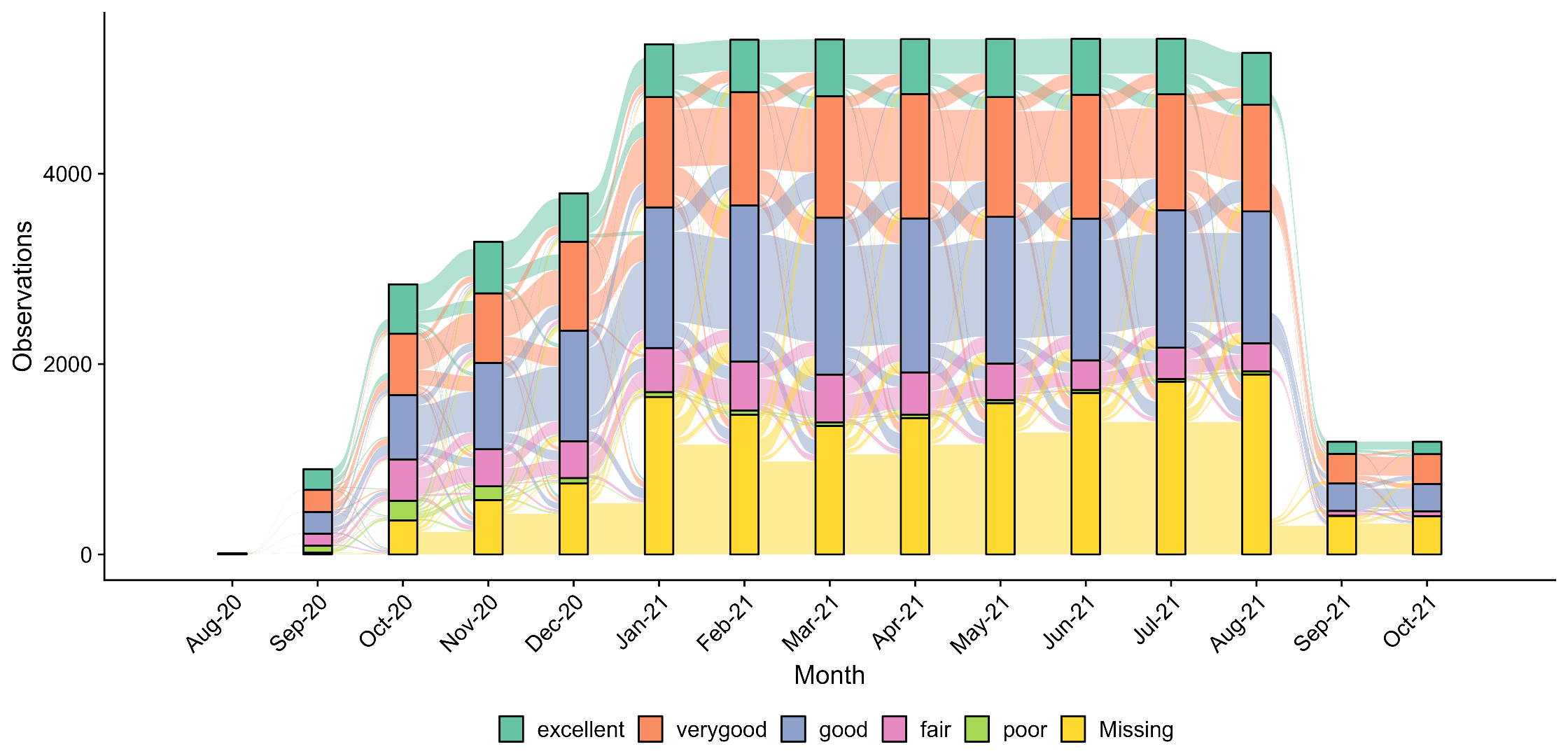


Supplementary figure S1: Alluvial plot of mental health scores (5-category ordinal) over the COVID-19 IMPACT study period. Bands between bars indicate the change in answer category from one month to the next.


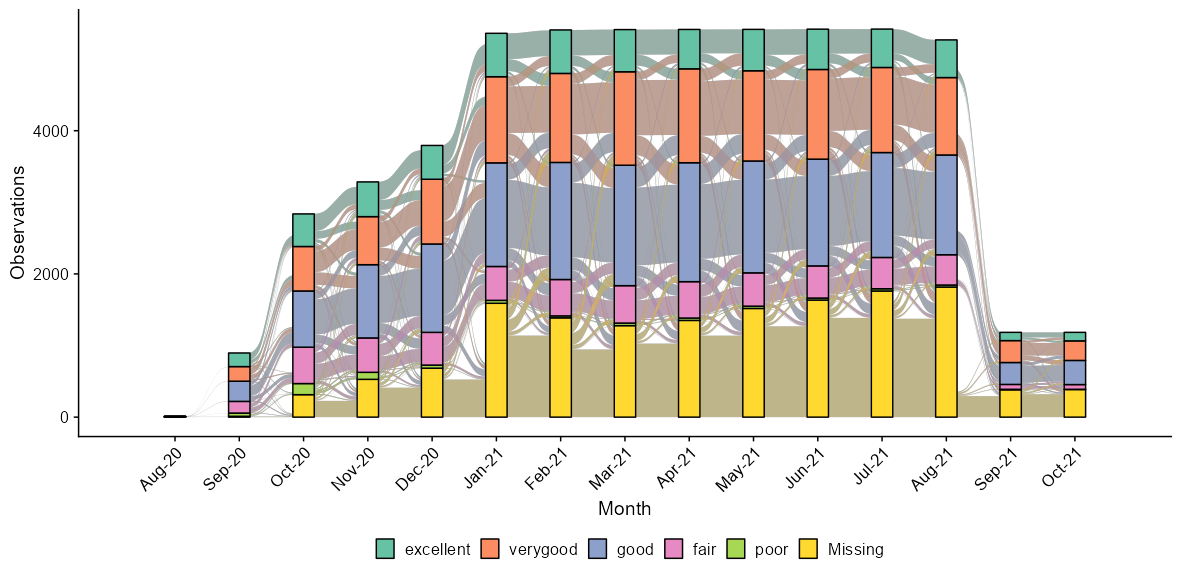


Supplementary figure S2: Alluvial plot of physical health scores (5-category ordinal) over the COVID-19 IMPACT study period. Bands between bars indicate the change in answer category from one month to the next.


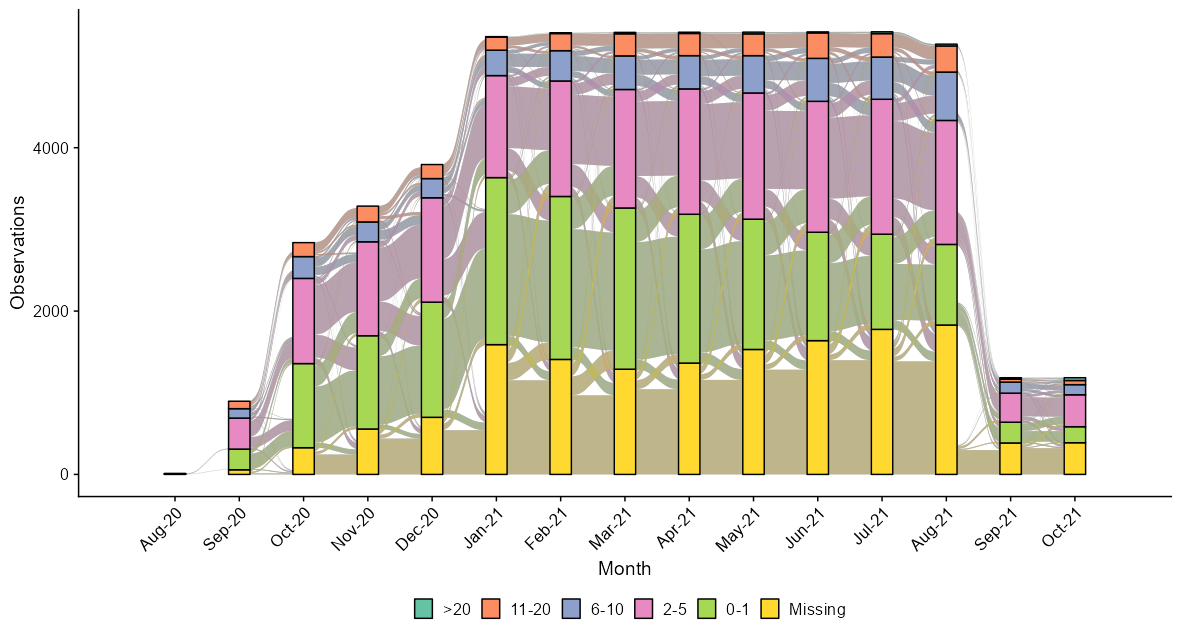


Supplementary figure S3: Alluvial plot of `n close interactions <1.5m`(5-category ordinal) over the COVID-19 IMPACT study period. Bands between bars indicate the change in answer category from one month to the next.


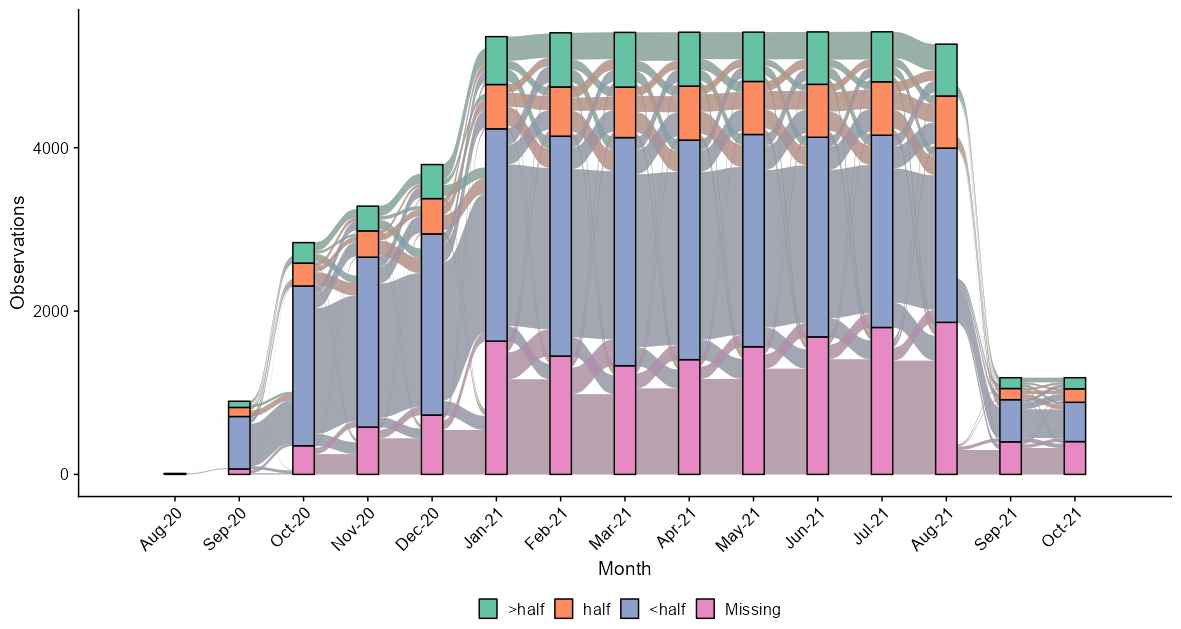


Supplementary figure S4: Alluvial plot of ‘close (<1.5m) interactions lasting >10m`(3-category ordinal) over the COVID-19 IMPACT study period. Bands between bars indicate the change in answer category from one month to the next.


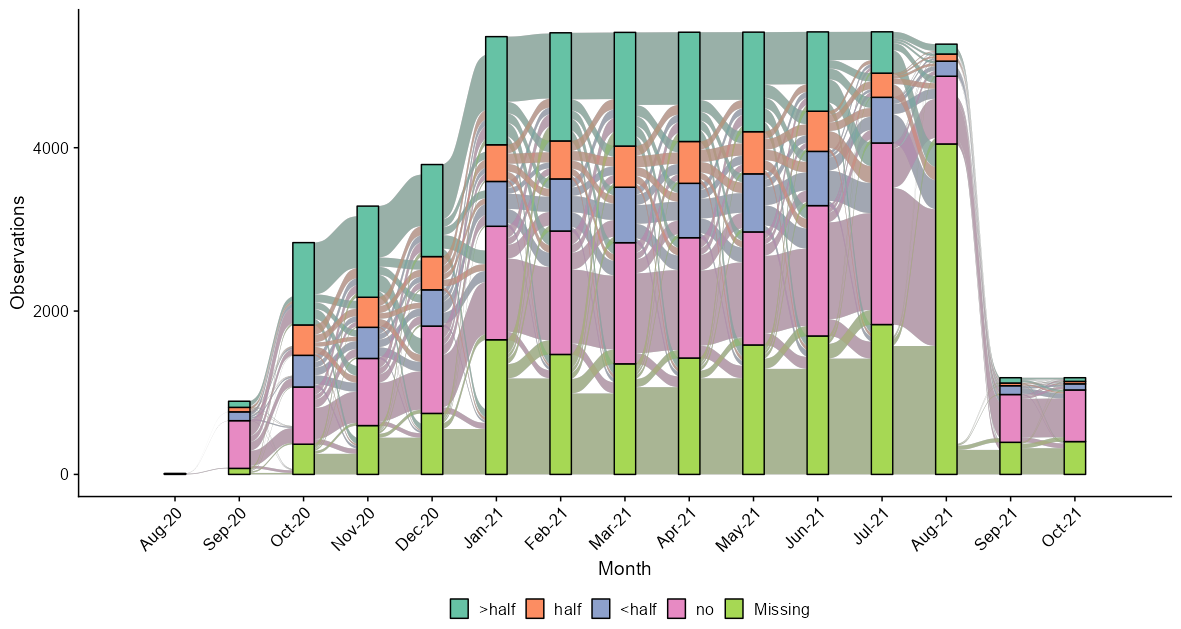


Supplementary figure S5: Alluvial plot of ‘personal protective equipment usage during close (<1.5m) contacts`(4-category ordinal) over the COVID-19 IMPACT study period. Bands between bars indicate the change in answer category from one month to the next.


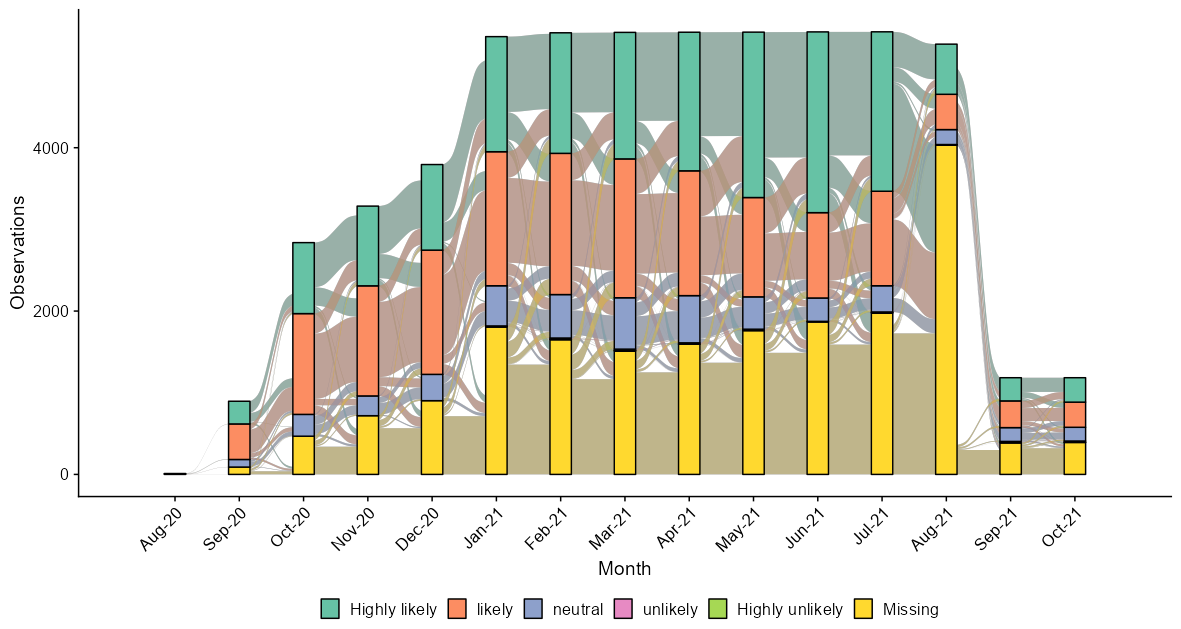


Supplementary figure S6: Alluvial plot of ‘Perceived probability of acquiring COVID-19`(5-category ordinal) over the COVID-19 IMPACT study period. Bands between bars indicate the change in answer category from one month to the next.


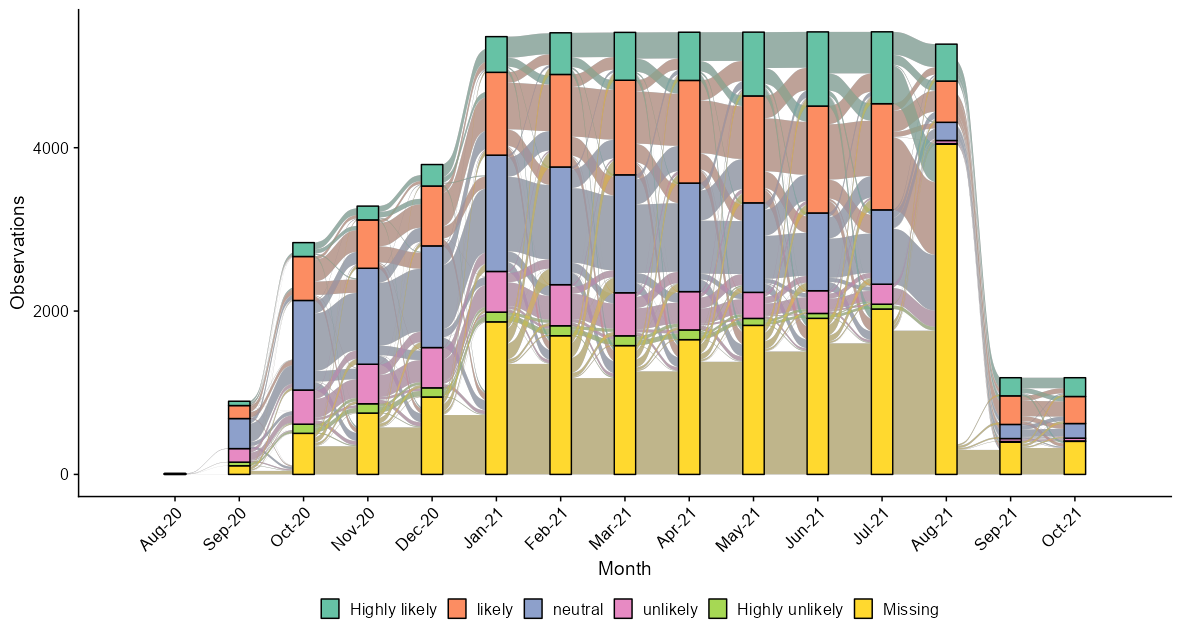


Supplementary figure S7: ‘perceived probability of becoming seriously ill from COVID-19’ (5-category ordinal) over the COVID-19 IMPACT study period. Bands between bars indicate the change in answer category from one month to the next.


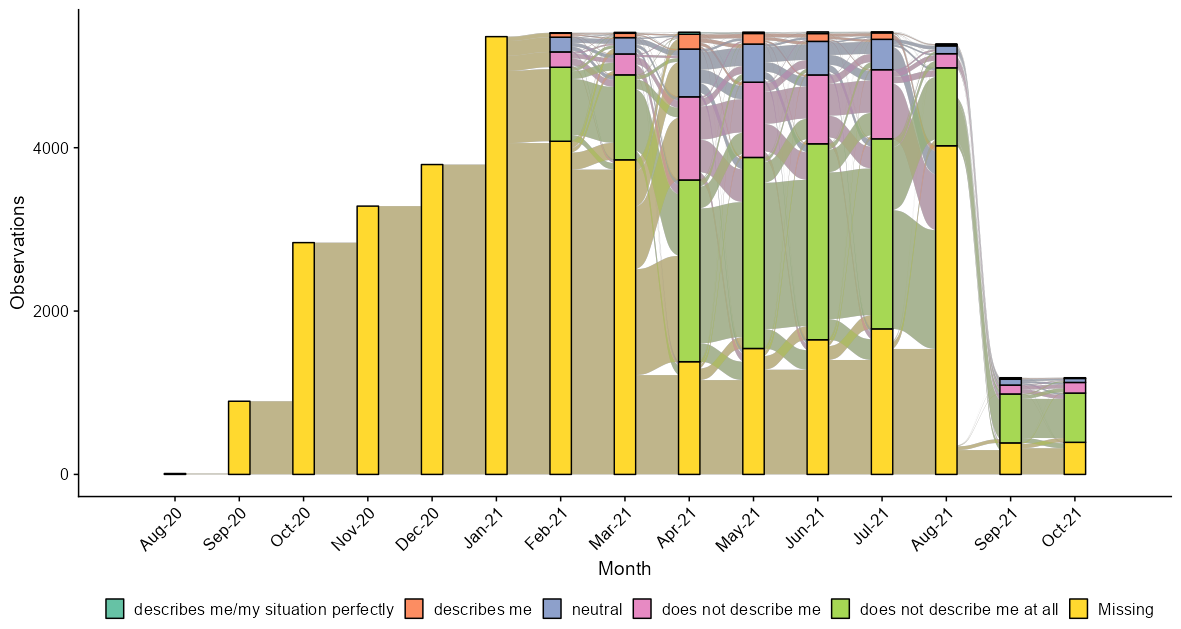


Supplementary figure S8: ‘I avoid healthcare in fear of acquiring COVID-19 in healthcare environments`(5-category ordinal) over the COVID-19 IMPACT study period. Bands between bars indicate the change in answer category from one month to the next. Note: this question was added in February 2021 (VGO) and April (AMIGO, PIAMA).


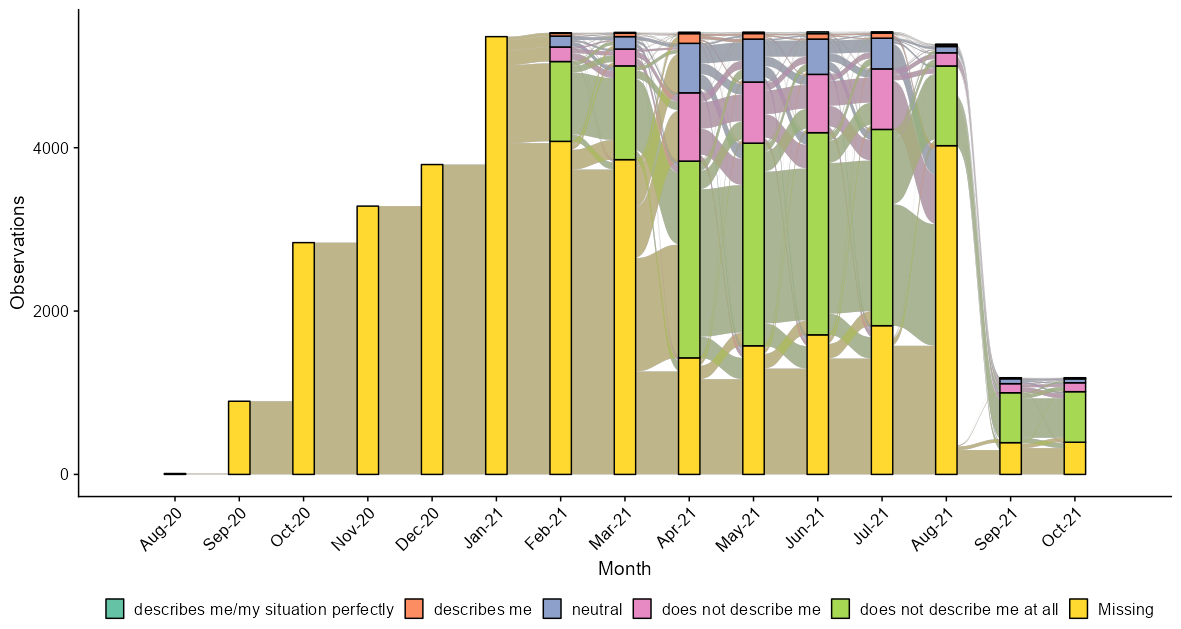


Supplementary figure S9: ‘I worry about my missed or postponed healthcare appointments`(5-category ordinal) over the COVID-19 IMPACT study period. Bands between bars indicate the change in answer category from one month to the next. Note: this question was added in February 2021 (VGO) and April (AMIGO, PIAMA).


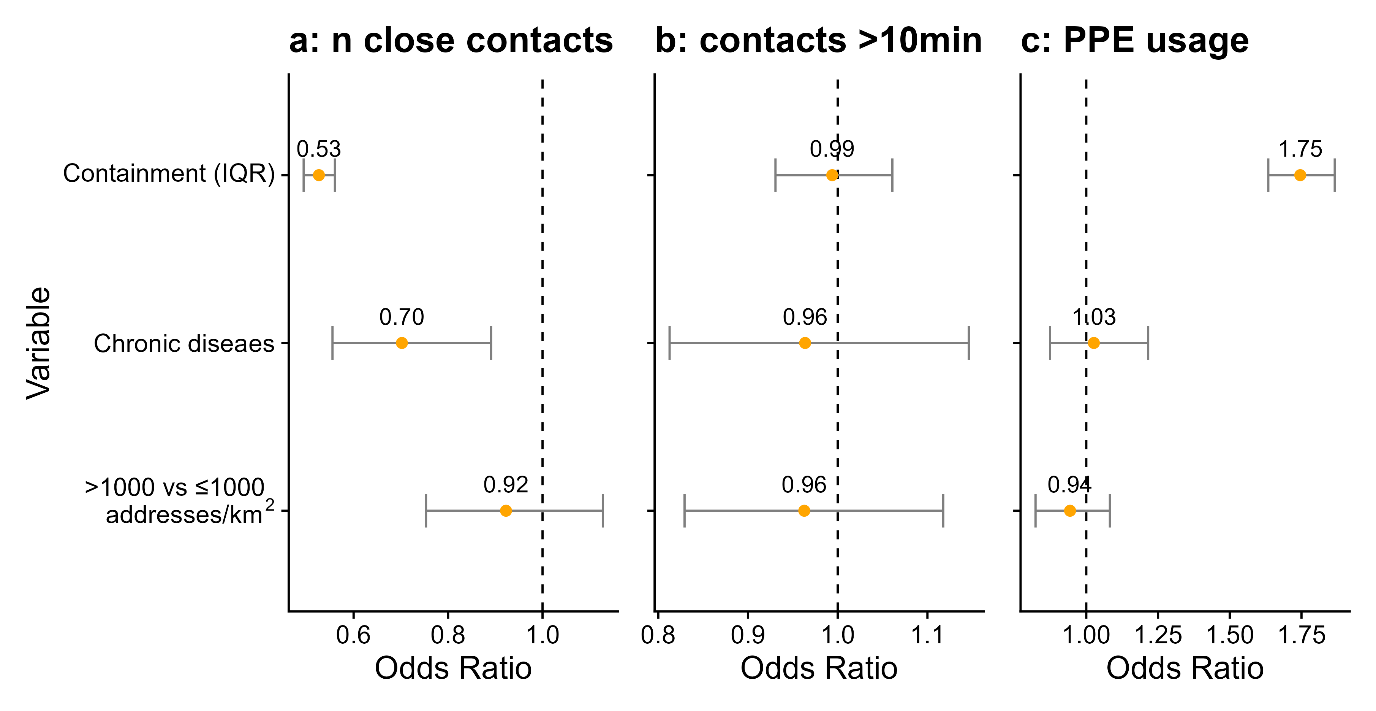


Supplementary figure S10 Bayesian multilevel main effect models for health protective behavior outcomes. a: Number of close contacts (<1.5m), b: Use of personal protective equipment (PPE) during close contacts, c: Close contact duration >10 minutes. Models were adjusted for age, sex, BMI, recruitment cohort and season.


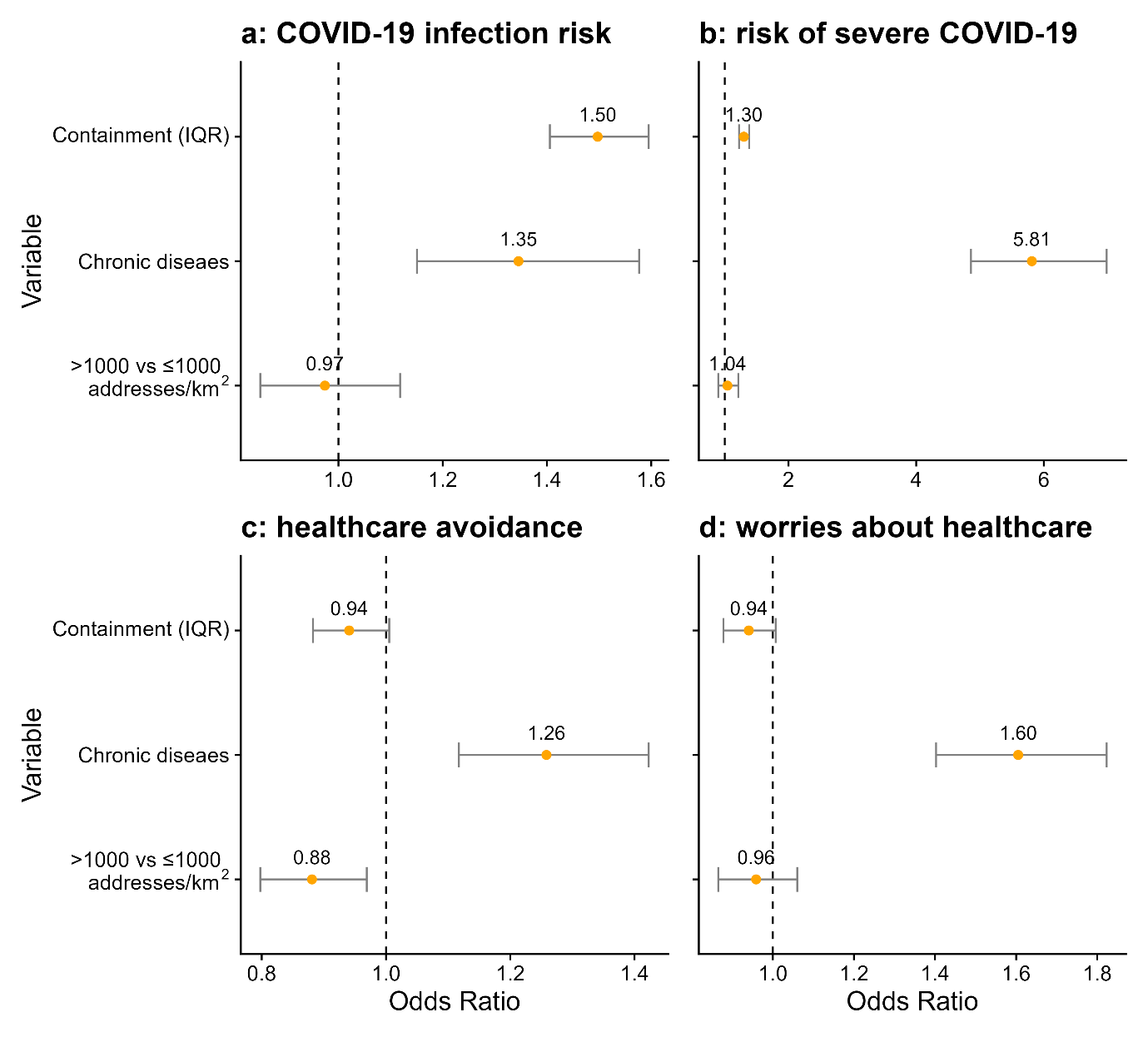


Supplementary figure S11 Bayesian multilevel main effect models for COVID-19 risk perception outcomes. a: Perceived risk of COVID-19 infection, b: Perceived risk of severe COVID-19, c: Healthcare avoidance in fear of acquiring COVID-19 in healthcare environments, d: Worry about missed or postponed healthcare appointments. Models were adjusted for age, sex, BMI, recruitment cohort and season.
